# Innovative microRNA quantification by qPCR

**DOI:** 10.1016/j.omtn.2023.02.012

**Published:** 2023-03-01

**Authors:** Minh Ngoc Le, Tuan Anh Nguyen

**Affiliations:** 1 Division of Life Science, The Hong Kong University of Science & Technology, Hong Kong, China

MicroRNAs (miRNAs) are essential regulators of gene expression in animals and plants. Accurate quantification of miRNA expression is crucial for understanding their role in cellular processes and human diseases. Qin et al. introduced SPLICER-qPCR (SplintR ligase-mediated ligation of complementary-pairing probes enhanced by RNase H-qPCR), a cost-effective and reliable tool for miRNA quantification from various sources, including clinical samples.^1^ SPLICER uses SplintR ligase and RNase H to enhance the measurement of complementary-pairing probes.

**Figure 1:**
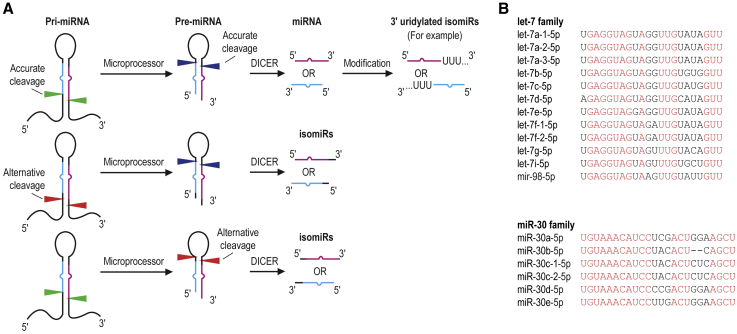
miRNA biogenesis and isoforms (isomiRs) (A) Multiple RNA sequences contain a miRNA sequence. The blue and pink strands represent the 5p and 3p miRNAs, respectively. The green and blue arrowheads indicate the cleavage sites of the Microprocessor and DICER enzymes, respectively. The red arrowheads indicate alternative cleavage sites of Microprocessor and DICER. (B) The human let-7 and miR-30 families. The similar nucleotides shared among all the members in each family are highlighted in red.

miRNA is a small RNA molecule consisting of approximately 22 nucleotides (nt) and has a 5′ monophosphate and a 3′ hydroxyl end. It is formed from its primary transcript, pri-miRNA, which is processed by Microprocessor to produce pre-miRNA and then cleaved by DICER to form the miRNA duplex. The mature miRNA is selected by Ago from a miRNA duplex^2^ (Figure 1A). Therefore, the sequence of miRNA is present in each form, including mature miRNA, pre-miRNA, and pri-miRNA. miRNAs in the same family have similar sequences, while different miRNA isoforms (isomiRs) may be produced by alternative processing or RNA modifications at the 3′ end^3^ (Figure 1A). For example, the human let-7 and miR-30 families contain 12 and 6 members, respectively, differing by 1–6 nt (Figure 1B). An effective miRNA quantification method should be capable of differentiating between the miRNA, its precursors, similar miRNAs, and isomiRs.

In the past 30 years, many methods for quantifying miRNAs have been developed. Northern blotting is one example. This uses a probe labeled with a radioactive isotope or nonradioactive tag that is complementary to the miRNA sequence.^4^ However, this method is inefficient for detecting low-abundance miRNAs, and it is time consuming and potentially harmful to the environment and our health. In addition, northern blotting is impractical for quantifying many miRNAs simultaneously. Microarrays can also be used to quantify miRNA, allowing the simultaneous profiling of numerous samples on a single chip.^4^ However, specialized equipment is required, and it is unfeasible to customize the system for a few miRNAs. Northern blotting and microarrays are also inefficient when discriminating between similar miRNAs or isomiRs.^4^ miRNAs can also be quantified by sequencing, and this method can distinguish miRNA sequences from their pre-/pri-miRNAs, similar miRNAs, and isomiRs.^4^ However, this method is time consuming and expensive, so it is impractical for quantifying a few miRNAs in different samples. In addition, miRNA sequencing does not provide a direct quantitative measurement of miRNA expression levels. Instead, expression levels are inferred from the sequencing reads.

qRT-PCR (reverse transcription quantitative polymerase chain reaction) is a commonly used and effective technique for miRNA quantification.^5^ The process involves RT of the miRNA into cDNA, followed by amplification and quantification of the cDNA through PCR. This method can be customized to quantify a small number to several hundred miRNAs, is capable of detecting low-abundance miRNAs due to the amplification step, and requires readily available reagents and equipment in most molecular biology laboratories.

**Figure 2:**
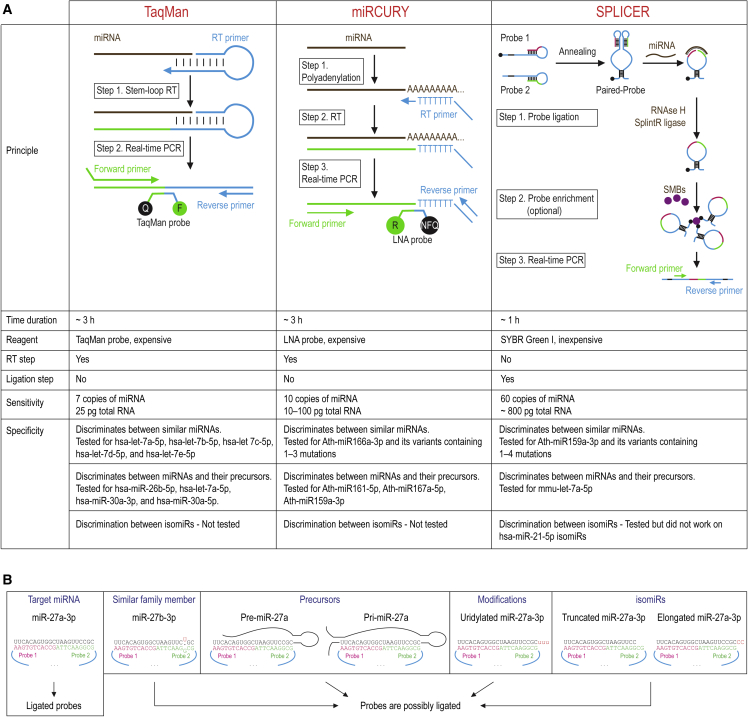
Quantification of miRNAs using qPCR methods (A) A comparison of TaqMan qPCR, miRCURY qPCR, and SPLICER-qPCR for miRNA quantification. The abbreviation RT stands for reverse transcription, PCR for polymerase chain reaction, and SMBs for streptavidin magnetic beads. (B) Factors that may impact the specificity of SPLICER-qPCR are shown, including the possibility of cross-reactivity with similar miRNAs in the same family, miRNA precursors, and isomiRs.

Over the past few decades, several qRT-PCR methods have been developed for miRNA quantification. However, the two most commonly used qRT-PCR methods for miRNA quantification are stem-loop qRT-PCR^6^ and poly(A) tailing^7^, available through Thermo Fisher Scientific’s TaqMan assay and Qiagen’s miRCURY qPCR assay, respectively (Figure 2A). The TaqMan qPCR uses a stem-loop RT primer to convert miRNA into cDNA, which is then amplified by two qPCR primers. This method can distinguish between similar miRNAs like the let-7 family members and identify miR-26b-5p, let-7a-5p, miR-30a-3p, and miR-30a-5p from their precursors. However, the ability for discrimination between isomiRs is unclear. The miRCURY qPCR, on the other hand, adds a poly(A) tail to the miRNA with poly(A) polymerase and then performs RT with a poly(T)-containing RT primer. The resulting cDNA is amplified with a miRNA-specific forward primer and a universal reverse primer that binds to the poly(T) adapter sequence. This method can differentiate between Ath-miR166a-3p and its variants containing 1–3 mutations. However, its ability to distinguish between miRNAs and their isomiRs is unknown. Both TaqMan qPCR and miRCURY qPCR are expensive as they require TaqMan probes and locked nucleic acid (LNA), respectively.

Qin et al. developed the SPLICER-qPCR method for quantifying miRNAs by designing a pair of single-stranded DNAs (ssDNAs) that specifically base pair with approximately half of the miRNA sequence to create an RNA/DNA hybrid^1^ (Figure 2A). The ssDNAs form a partially double-stranded DNA (dsDNA) structure that serves as a binding platform for interaction with miRNA. Subsequently, the ssDNAs are ligated together using SplintR ligase. To prevent ligating the ssDNAs without miRNAs, a stem-loop structure is adopted, keeping the ligatable ends in base pairs in each ssDNA and preventing nonspecific binding with pri-/pre-miRNAs and nontarget miRNAs. The ssDNA probes are equipped with two universal DNA regions that are capable of binding to two common qPCR primers. This feature could potentially allow for the quantification of multiple miRNAs using a single set of primers. To improve specificity and sensitivity, the SPLICER-qPCR method includes a probe enrichment step using magnetic beads to eliminate background interference.

The SPLICER-qPCR method has been proven to accurately measure miRNAs from various sources, like fresh broccoli, mouse tissues, and rat peripheral circulation samples, with sensitivity equal to or better than the miRCURY and TaqMan qPCR methods. SPLICER-qPCR is therefore a promising option for miRNA quantification in research and molecular diagnosis. However, there are areas for improvement (Figure 2B). SPLICER-qPCR can differentiate between variants of Ath-miR159a-3p with 1–4 mutations, but its ability to distinguish similar miRNAs in the same family is unknown. SPLICER-qPCR shows a significant difference between pre-let-7a and let-7a-5p, but further validation with more miRNAs is needed to confirm its ability to discriminate miRNAs from their pri/pre-miRNAs. Additionally, SPLICER-qPCR has the ability to amplify isomiRs of miR-21-5p, but more investigation is needed to determine its precision in discriminating isomiRs of miR-21-5p and also other miRNAs.

qRT-PCR is a valuable method for investigating miRNA expression due to its ease of use. Despite limitations, different qRT-PCR methods have been widely used in research for many years. To improve its utility, future studies should aim to increase its sensitivity and efficiency for miRNA quantification, as well as address the three key challenges of differentiating miRNA from its family members, precursors, and isomiRs.
